# Integrating Physiology and Multi-Omics Reveals Mechanisms Underlying Sperm Activation and Movement in Mandarin Fish (*Siniperca chuatsi*)

**DOI:** 10.3390/ani16172762

**Published:** 2026-09-02

**Authors:** Qinghua Wang, Yuxin Zhang, Zhong Huang, Weiwei Zhang, Yingxin Wu, Jiajie Li, Yizheng Zhang, Lu Li, Zhiming Zhu, Zining Meng

**Affiliations:** 1 School of Life Sciences, State Key Laboratory of Biocontrol/Guangdong Core Germplasm Bank for Marine Economic Animals, Southern Marine Science and Engineering Guangdong Laboratory (Zhuhai)/Guangdong Provincial Key Laboratory of Aquatic Economic Animals, Sun Yat-sen University, Guangzhou 510275, China; wangqh55@mail2.sysu.edu.cn (Q.W.); zhangyx558@mail2.sysu.edu.cn (Y.Z.); zhangww79@mail2.sysu.edu.cn (W.Z.); wuyx328@mail2.sysu.edu.cn (Y.W.); lijj379@mail2.sysu.edu.cn (J.L.); zhangyzh253@mail2.sysu.edu.cn (Y.Z.); lilu95@mail2.sysu.edu.cn (L.L.); 2 Shenzhen Base of South China Sea Fisheries Research Institute, Chinese Academy of Fishery Sciences, Shenzhen 518121, China; huangzhongnhs@163.com; 3 China-ASEAN Belt and Road Joint Laboratory on Mariculture Technology, Guangzhou 510275, China

**Keywords:** *Siniperca chuatsi*, physiology, transcriptomics and proteomics, osmolality and ion, sperm activation and movement

## Abstract

Sperm activation and movement are essential in freshwater fishes, where limited energy reserves and a short motility duration require rapid ATP-driven motility regulated by osmolality and ions. However, the physiological and molecular mechanisms underlying osmolality and ion regulation in mandarin fish (*Siniperca chuatsi*) remain unclear, and ions in aquaculture systems may exert additional effects. Here, we integrated physiology with transcriptomic and proteomic analyses to characterize the effects of osmolality and ions on sperm motility and identify key genes, proteins, and regulatory networks involved in sperm activation and movement. Low osmolality (<50 mOsm/kg) promoted sperm activation, with sperm motility progressively decreasing as osmolality increased. Although relatively high osmolality supported motility maintenance, the initial motility was comparatively low, indicating the limited energy reserves of freshwater fish spermatozoa. Na^+^ and K^+^ activation media enhanced motility, whereas Ca^2+^ supplementation suppressed activation. Ca^2+^ levels below a 1:500 molar ratio (<0.06 mM) did not significantly affect sperm motility, indicating the importance of limiting Ca^2+^ exposure during fertilization. Multi-omics analyses identified six differentially expressed genes, including *grin3bb*, *cacna1c*, and *chrna7*, and 35 differentially expressed proteins, including ATP1B, PRKCB, and CPT1A, as key molecules associated with Na^+^, K^+^, and Ca^2+^ signaling and energy metabolism. The calcium signaling pathway, cAMP signaling pathway, and oxidative phosphorylation were significantly enriched in KEGG enrichment analysis, while Gene Ontology (GO) enrichment analysis identified ion signaling and energy metabolism, including ion transport, the ATP metabolic process, and the glycolytic process. Our findings provide a putative regulatory network for sperm activation and movement in mandarin fish and a basis for optimizing artificial fertilization.

## Simple Summary

Sperm activation and movement are critical for successful fertilization in mandarin fish, but the effects of osmolality and ions on these processes remain poorly understood. In this study, we combined physiological experiments with transcriptomic and proteomic analyses to investigate how osmolality and key ions regulate sperm motility. The results showed that low osmolality promoted sperm activation, whereas sperm motility gradually decreased as osmolality increased. Na^+^ and K^+^ improved sperm motility, while Ca^2+^ inhibited sperm activation, especially at higher concentrations. When Ca^2+^ was maintained below a 1:500 molar ratio, sperm motility was not significantly affected, suggesting that Ca^2+^ exposure should be minimized during artificial fertilization. Multi-omics analyses identified several important genes and proteins related to ion transport, calcium signaling, and energy metabolism. These results suggest that sperm activation and movement in mandarin fish is regulated by coordinated ion signaling and energy metabolism. This study provides new insights into the molecular regulation of sperm motility and offers practical guidance for improving artificial fertilization techniques in mandarin fish.

## 1. Introduction

Sperm activation and movement are crucial in freshwater fishes, due to lower energy reserves and shorter motility durations compared with marine species, requiring rapid fertilization through ATP-fueled bursts of motility modulated by osmolality and ions [1,2]. In most freshwater fishes, low osmolarity (<300 mOsm/kg) triggers sperm activation, while high osmolarity (>400 mOsm/kg) promotes sperm motility in marine species [1,3]. After activation, marine fish spermatozoa, supported by greater energy reserves, often sustain motility for more than 300 s, whereas most freshwater fish spermatozoa contain comparatively lower energy stores and typically remain motile for less than 60 s [2,4]. Na^+^ plays a critical role in stimulating sperm activation and maintaining motility in both marine and freshwater fish species [5,6]. K^+^, however, exerts dual effects on sperm activation, inhibiting motility in some freshwater fishes but inducing membrane depolarization and motility initiation in marine species [6,7]. Sperm activation can be triggered without extracellular Ca^2+^ in several teleosts [8,9], whereas extracellular Ca^2+^ is required for motility initiation in some fish [10], and in certain freshwater fish, the addition of Ca^2+^ can even inhibit motility [6,11]. Hence, the precise regulation of osmolarity and ion composition is essential for sperm activation in freshwater fishes characterized by low energy stores and short motility durations.

With the rapid development of high-throughput sequencing technologies, transcriptomics and proteomics provide practical approaches to investigate gene expression and protein function in sperm [12,13,14], advancing our understanding of the molecular basis of fish sperm activation. Transcriptomic profiling of gilthead seabream (*Sparus aurata*) during spermiogenesis identifies gene expression patterns primarily associated with signaling, metabolism, ion transport, and cytoskeletal regulation, highlighting the broad molecular changes accompanying sperm differentiation and maturation [15]. Transcriptomics reveals that fetal bovine serum enhances sperm motility in black rockfish (*Sebastes schlegelii*) by promoting sperm capacitation, orchestrated via cAMP-SRC-PKA, cGMP-PKG, and phospholipase D signaling, with *hsp90aa1* and *cxcr4* playing pivotal roles in flagellar integrity and motility [16]. Furthermore, proteomic studies uncover proteins that serve as biomarkers of sperm quality and freezability, enabling the precise management of artificial reproduction and enhancement of cryopreservation strategies [17]. Fish spermatozoa possess a wide spectrum of proteins regulating signaling, intracellular transport, cytoskeletal dynamics, protein turnover, and stress responses, alongside metabolic pathways such as glycolysis, lipid metabolism, and the TCA cycle, collectively sustaining motility and function [18,19,20,21]. Taken together, these findings underscore the considerable potential of omics approaches to dissect the molecular networks underlying fish sperm activation.

Mandarin fish (*Siniperca chuatsi*), commonly known as osmanthus or Chinese perch, is a benthic piscivore of the Serranidae family, widely distributed in East Asian freshwater habitats, including China, Korea, and Vietnam [22,23]. Its favorable production traits, including the absence of intermuscular bones, excellent meat quality, and high nutritional value, have made it a premium food fish with substantial economic importance in China [24]. Since artificial propagation technologies were successfully developed in the late 1980s, aquaculture production has increased dramatically. Sustained market demand has since established the species as one of the most valuable freshwater aquaculture commodities in China, with production surpassing 600,000 tons in 2025 [25]. However, the physiological and molecular mechanisms underlying the effects of osmolality and ions on sperm motility in mandarin fish remain unclear, and ions in aquaculture systems may exert additional effects. Studies of gene and protein expression associated with sperm activation and movement are also limited. Here, we integrated sperm motility assays with transcriptomic and proteomic analyses to investigate mechanisms regulating sperm activation and movement. We characterized the effects of osmolality and ions on sperm motility and identified key genes, proteins, and regulatory networks. Our findings provide a physiological and molecular framework for sperm activation and movement in mandarin fish and offer a basis for improving artificial fertilization strategies.

## 2. Materials and Methods

### 2.1. Ethics Statement, Broodstock Management, and Sperm Collection

All experimental procedures were approved by the Institutional Animal Care and Use Committee of the School of Life Sciences, Sun Yat-sen University. Experiments were carried out during the reproductive season of mandarin fish, from April to June 2026. Sexually mature males (body length 27.66 ± 1.48 cm, body weight 658 ± 106 g, age 13 ± 1 month, *n* = 24) were obtained from Liangshi Aquatic Seed Industry Co., Ltd., Foshan, China. Broodstock were maintained in a 5 m × 5 m × 2 m concrete tank with aerated, flow-through freshwater (temperature 24.69 ± 0.88 °C, pH 8.15 ± 0.42, dissolved oxygen 7.82 ± 0.44 mg/L, ammonia nitrogen 0.213 ± 0.047 mg/L, and nitrite nitrogen 0.0029 ± 0.0009 mg/L) under a natural photoperiod (13 h light/11 h dark). Following 5 min of eugenol anesthesia, sperm were collected via abdominal massage and stored at 4 °C in 1.5 mL RNase-free tubes. Only uncontaminated samples (free of blood, urine, or feces) with initial motility exceeding 85% were used for physiological, transcriptomic, and proteomic analyses.

### 2.2. Physiological Analysis

Sperm from 24 males was used to assess the effects of osmolality and ionic composition on motility after activation. Each treatment was carried out in at least three replicates. For motility measurement, 0.5 μL of sperm was combined with 50 μL of activation solution and gently mixed to ensure uniform activation. The suspension was immediately examined using a UB200i microscope equipped with an ISAS 782C color CCD camera (Proiser R + D S.L., Paterna, Spain). Motility was quantified using the Integrated Semen Analysis System (ISAS v1.2; Proiser R + D S.L., Paterna, Spain) after sperm activation 10 s, 20 s, and 30 s, corresponding to early activation, motility maintenance, and motility decline. In filtered freshwater, motility decreased from 89.10 ± 2.83% at activation 10 s to 55.62 ± 5.27% at 20 s and 6.54 ± 3.25% at 30 s. Activation solutions were prepared from sucrose, artificial sea salt (mainly containing Na^+^, Mg^2+^, Ca^2+^, K^+^, and Sr^2+^), NaCl, KCl, and CaCl_2_ (Yier, Guangzhou, China). All reagents were dissolved in distilled water with the pH adjusted to 7.8–8.2 (FE20K Plus, Mettler Toledo, Shanghai, China). Preliminary experiments indicated that sperm activation occurred across 0–300 mOsm/kg, and thus 500 mOsm/kg stock solutions were used to generate activation solutions at 50 mOsm/kg intervals. The 500 mOsm/kg stock solutions included 22.5 g/L artificial sea salt, 500 mM sucrose, 250 mM NaCl, 250 mM KCl, and 167 mM CaCl_2_, respectively. Sperm samples were centrifuged twice at 12,000× *g* for 5 min at 4 °C to isolate seminal plasma, and the osmolality of seminal plasma from mandarin fish (367–380 mOsm/kg) was measured using an Osmomat 030 cryoscopic osmometer (Gonotec, Berlin, Germany).

#### 2.2.1. Experiment 1: Effect of Osmolality

The impact of osmolality on sperm motility was evaluated by activating samples in sucrose and artificial sea salt solutions ranging from 0 to 300 mOsm/kg, with motility recorded at 10, 20, and 30 s post-activation. Sucrose was used to assess osmotic effects without ions, while artificial sea salt provided a multi-ionic reference.

#### 2.2.2. Experiment 2: Effect of Ions

To evaluate individual ion effects, sperm were activated in NaCl and KCl solutions across the same osmolality range, with motility recorded at 10, 20, and 30 s post-activation. Given the inhibitory role of Ca^2+^, motility in CaCl_2_ activation media was assessed at 10 and 20 s post-activation.

#### 2.2.3. Experiment 3: Effect of Combined Ions, pH

Based Experiments 1 and 2, 50 mOsm/kg was determined as the optimal osmolality for activation. To explore the combined effect of Na^+^ and K^+^, sperm were activated at molar ratios of 7:1, 5:1, 3:1, 1:1, 1:3, 1:5, and 1:7, with motility measured at 10 s post-activation. Due to the inhibitory role of Ca^2+^, the effects of CaCl_2_ + NaCl and CaCl_2_ + KCl were further evaluated using CaCl_2_:NaCl/KCl at molar ratios of 1:10, 1:20, 1:50, 1:100, 1:200, 1:500, and 1:1000. Under the same osmotic condition, using NaCl solutions, sperm motility was further evaluated across a pH gradient of 4–11 at 10 s post-activation.

### 2.3. Transcriptomics Analysis

#### 2.3.1. Omics Sample Collection

Among the 24 males used for sperm motility analysis, 12 were further selected for omics analyses. Six males were used for transcriptomic analysis and the remaining six for proteomic analysis; within each analysis, three males were assigned to the activated sperm (AS) group and three to the fresh sperm (FS) group. For the AS groups, sperm were activated in naturally filtered freshwater for 60 s. This timeframe included all stages of early activation, motility maintenance, and motility decline, ensuring comprehensive transcriptomes and proteomes being captured. Unactivated fresh sperm samples were defined as controls. Sperm pellets were obtained by centrifugation at 12,000× *g* for 5 min at 4 °C, rapidly frozen in liquid nitrogen, and stored at −80 °C until library construction.

#### 2.3.2. RNA Extraction, cDNA Library Construction, and Transcriptome Sequencing

Total RNA was extracted from sperm using TRIzol reagent (Invitrogen, Carlsbad, CA, USA) following the manufacturer’s protocol. RNA integrity and quality were evaluated using an Agilent 2100 Bioanalyzer (Agilent Technologies, Palo Alto, CA, USA). Polyadenylated mRNA was enriched using oligo(dT) magnetic beads and fragmented. First-strand cDNA was synthesized with random primers, followed by second-strand synthesis. Subsequently, the cDNA fragments were purified, end-repaired, A-tailed, and ligated to sequencing adapters. Libraries were sequenced on an Illumina NovaSeq 6000 platform (Illumina, San Diego, CA, USA) by Novogene Co., Ltd. (Beijing, China).

#### 2.3.3. Quality Control, Assembly of Transcripts, and Differential Expression Analysis

Raw sequencing reads were processed using fastp v.0.24.0 [26] to remove adapters, poly-N sequences, and low-quality reads. Read quality was assessed using Q20, Q30, and GC content. Clean reads were aligned to the *S. chuatsi* reference genome (GCA_020085105.1) using Hisat v.2.2.1 [27], and transcripts were assembled with StringTie v.3.0.1 [28]. Unannotated transcripts were classified as novel genes. Gene expression was quantified in fragments per kilobase of transcript per million mapped reads (FPKM) using RSEM v.1.3.3 [29]. Differential expression was assessed with DESeq2 v.1.42.0 [30], with a false discovery rate (*FDR*) < 0.05 and |log2 (fold change)| > 1 used to define differentially expressed genes (DEGs).

### 2.4. Proteomics Analysis

#### 2.4.1. Total Protein Extraction, Protein Quality Assessment, and Trypsin Digestion

Omics samples were prepared as described in Section 2.3.1. Sperm samples were lysed in lysis buffer (6 M urea, 100 mM TEAB, pH 8.5) and sonicated on ice for 5 min. The lysates were centrifuged at 12,000× *g* for 15 min at 4 °C, and supernatants were collected. Proteins were reduced with DTT (Sigma, St. Louis, MO, USA) at 56 °C for 1 h and alkylated with iodoacetamide for 1 h in the dark at room temperature. Reaction was terminated by incubation on ice for 2 min. Protein concentrations were measured using the Bradford assay (Beyotime, Shanghai, China) with BSA standards. Equal amounts of protein (20 μg) were separated by 12% SDS-PAGE and visualized by Coomassie Brilliant Blue R-250 staining. Protein samples were diluted to 100 μL and digested with trypsin at 37 °C for 4 h. Digests were acidified to pH < 3 with formic acid, centrifuged, desalted using C18 columns, and eluted with 70% acetonitrile containing 0.1% formic acid. Eluates were lyophilized before further analysis.

#### 2.4.2. Liquid Chromatography–Tandem Mass Spectrometry (LC-MS/MS) Analysis

Peptides were dissolved in solvent A (99.9% water, 0.1% formic acid), centrifuged at 14,000× *g* for 20 min at 4 °C, and 200 ng of peptides was injected for LC–MS analysis. Peptides were separated using a Vanquish Neo UHPLC system (Thermo Fisher Scientific, Waltham, MA, USA) equipped with a C18 pre-column (174500, 5 mm × 300 µm, 5 µm) and a C18 analytical column (ES906, PepMap™ Neo UHPLC, 150 µm × 15 cm, 2 µm) maintained at 50 °C. MS analysis was performed on an Orbitrap Astral mass spectrometer coupled to an Easy-Spray ESI source in data-independent acquisition (DIA) mode. Full MS scans were acquired over m/z 380–980 at 240,000 resolution with 2 Th isolation windows across 300 DIA segments. MS2 spectra were collected over m/z 150–2000 at Astral resolution 80,000 with NCE 25% and a maximum injection time of 3 ms.

#### 2.4.3. Protein Identification and Quantitation

Raw MS data were analyzed using DIA-NN against the *S. chuatsi* protein database. Precursor and fragment mass errors were automatically corrected. Carbamidomethylation of cysteine was used as a fixed modification, while N-terminal methionine excision was included as a variable modification, allowing up to two missed cleavages. Peptide and protein identifications were filtered using Global Q-Value and PG Q-Value thresholds of 0.01. Proteins with *p* < 0.05 and fold change (FC) > 1.2 or <0.83 were identified as differentially expressed proteins (DEPs).

### 2.5. Bioinformatics Analysis

Principal component analysis (PCA) and Pearson correlation analysis were performed in R v.4.4.3 to evaluate relationships among samples and the reproducibility of biological replicates. DEGs and DEPs were annotated against Kyoto Encyclopedia of Genes and Genomes (KEGG) and Gene Ontology (GO) databases for functional and pathway analysis, with significant enrichment defined as *p* < 0.05. To identify highly connected hub proteins, DEGs and DEPs were mapped to STRING v12.0 [31] to generate protein–protein interaction (PPI) networks. Critical DEGs and DEPs involved in key pathways were integrated into mRNA-pathway-protein regulatory network, which was visualized using Cytoscape v3.10.1 [32].

### 2.6. Statistical Analysis

Statistical analyses were performed using SPSS v.22.0 (SPSS Inc., Chicago, IL, USA). Differences between groups were assessed using Student’s T-test or one-way ANOVA followed by Duncan’s multiple range test. Each treatment was performed in at least three replicates. Data are presented as mean ± standard deviation (SD), and differences were considered statistically significant at *p* < 0.05. Graphical visualization was performed using GraphPad Prism v.9.5.0 (GraphPad Software, San Diego, CA, USA).

## 3. Results

### 3.1. Physiological Analysis

#### 3.1.1. Effect of Osmolality

**Figure 1:**
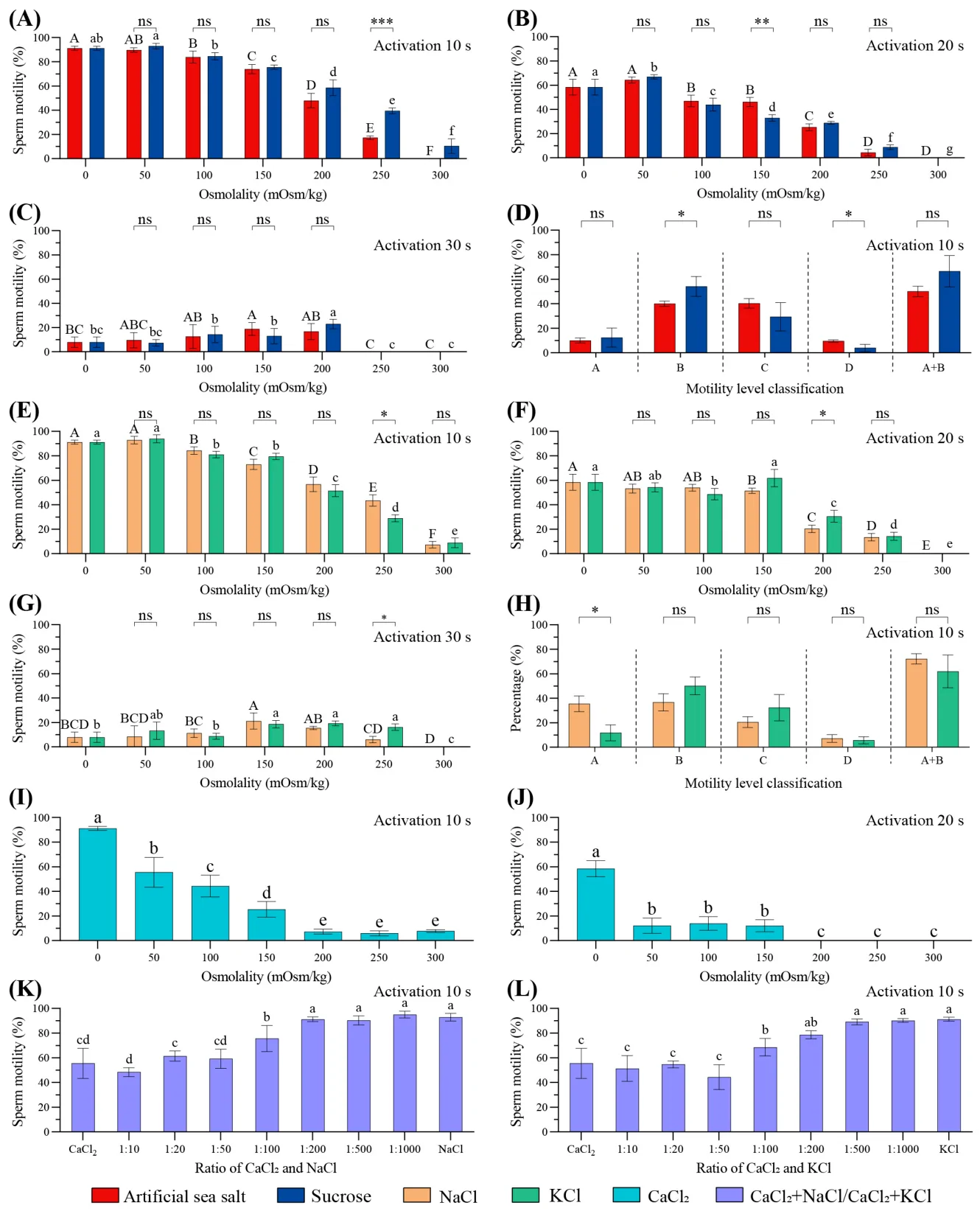
Physiological analysis. (**A**–**D**) Effects of osmolality. Artificial sea salt and sucrose solutions were used to evaluate the effects of osmolality on sperm motility. Motility was recorded at 10 s (**A**), 20 s (**B**), and 30 s (**C**) post-activation across an osmolality range of 0–300 mOsm/kg. Uppercase and lowercase letters denote significant differences among osmolalities within artificial sea salt and sucrose treatments, respectively (*p* < 0.05). Significance: ns, *p* > 0.05; *, *p* < 0.05; **, *p* < 0.01; ***, *p* < 0.001. (**D**) Motility level classification at 50 mOsm/kg and 10 s post-activation. (**E**–**J**) Effect of ions. Sperm motility in NaCl and KCl activation media was recorded at 10 s (**E**), 20 s (**F**), and 30 s (**G**) post-activation across different osmolalities. Uppercase and lowercase letters indicate significant differences within NaCl and KCl treatments, respectively (*p* < 0.05). Significance: ns, *p* > 0.05; *, *p* < 0.05. (**H**) Motility level classification at 50 mOsm/kg and 10 s post-activation in NaCl and KCl. Under CaCl_2_ activation conditions, motility was measured at 10 s (**I**) and 20 s (**J**) post-activation across different osmolalities. Lowercase letters indicate significant differences within CaCl_2_ treatments (*p* < 0.05). (**K**–**L**) Effect of combined ions. Sperm motility at 50 mOsm/kg and 10 s post-activation under varying CaCl_2_ and NaCl combinations (**K**) or CaCl_2_ and KCl combinations (**L**). Different lowercase letters represent significant differences among treatments (*p* < 0.05). Each treatment was performed in at least three replicates.

Sperm showed high motility (>70%) at low osmolalities (0–150 mOsm/kg) in both artificial sea salt and sucrose solutions and declined as osmolality increased (Figure 1A–C; Supplementary Data S1). Overall, sperm activation was predominantly regulated by osmolality, with ionic composition playing a modulatory effect. At 10 s post-activation, high motility was maintained at 0–50 mOsm/kg in both media, whereas motility decreased progressively with increasing osmolality when osmolality exceeded 100 mOsm/kg (Figure 1A). At 20 s post-activation, relatively high motility persisted at 0–50 mOsm/kg in artificial sea salt and at 50 mOsm/kg in sucrose (Figure 1B). At 30 s post-activation, sperm motility decreased to below 30% in both artificial sea salt and sucrose media, although relatively higher motility was observed at 200 mOsm/kg (Figure 1C). At 250 mOsm/kg and 10 s post-activation, sucrose elicited higher motility than artificial sea salt; at 150 mOsm/kg and 20 s post-activation, motility in artificial sea salt was higher than in sucrose (Figure 1A–C). At 50 mOsm/kg, sucrose significantly increased the proportion of rapidly motile (B level) sperm and decreased weakly motile (D level) sperm at 10 s post-activation (*p* < 0.05) (Figure 1D).

#### 3.1.2. Effect of Ions

The influence of Na^+^, K^+^, and Ca^2+^ on sperm motility was evaluated. At 10 s post-activation, high motility was maintained at 0–50 mOsm/kg in both NaCl and KCl solutions (Figure 1E). At 20 s post-activation, NaCl supported high motility at 0–100 mOsm/kg, while KCl maintained high motility at 0–50 and 150 mOsm/kg (Figure 1F). At 30 s post-activation, sperm motility decreased to below 40% in both NaCl and KCl media, although relatively higher motility was observed at 150–200 mOsm/kg (Figure 1G). Under the same osmotic conditions, NaCl promoted higher motility than KCl at 250 mOsm/kg and 10 s post-activation (Figure 1E), while KCl promoted higher motility than NaCl at 200 mOsm/kg and 20 s post-activation and at 250 mOsm/kg and 30 s post-activation (*p* < 0.05) (Figure 1F,G). At 50 mOsm/kg and 10 s post-activation, NaCl significantly increased the proportion of rapidly motile (A level) sperm compared with KCl (*p* < 0.05) (Figure 1H). Notably, CaCl_2_ activation strongly reduced motility at both 10 s and 20 s post-activation (Figure 1I,J), indicating an inhibitory effect of Ca^2+^ on sperm motility.

#### 3.1.3. Effect of Combined Ions, pH

Because high motility was consistently observed at 50 mOsm/kg in artificial sea salt, sucrose, NaCl, and KCl, this was selected as the optimal osmolality. Motility did not differ significantly among NaCl:KCl ratios at 50 mOsm/kg (Figure S1A). Motility classification analysis revealed that specific ion ratios improved sperm quality. At 10 s post-activation, NaCl:KCl ratios of 7:1, 1:5, and 1:7 significantly increased the proportion of A level sperm (*p* < 0.05) (Figure S1B), indicating a synergistic role of Na^+^ and K^+^.

The effects of combined ions (CaCl_2_ + NaCl and CaCl_2_ + KCl) were further evaluated. Sperm motility increased progressively as the proportion of Ca^2+^ in the activation media decreased. At 10 s post-activation, motility in NaCl solution and in CaCl_2_:NaCl ratios of 1:200, 1:500, and 1:1000 was significantly higher than that at Ca^2+^ ratios (1:10–1:100) and in CaCl_2_ solution (Figure 1K). A similar trend was observed in CaCl_2_:KCl treatments, where 1:500 and 1:1000 ratios and KCl solutions yielded higher motility than other Ca^2+^ ratios (1:10–1:100) and CaCl_2_ solution (Figure 1L). Overall, Ca^2+^ levels below a 1:500 molar ratio (<0.06 mM) did not significantly affect sperm motility.

Peak motility was observed at pH 8, 10 s post-activation using NaCl activation medium (50 mOsm/kg) (Figure S2A). Further classification across pH 5–10 showed that pH 8 significantly increased the proportion of rapidly motile sperm (B and A + B levels) while reducing weakly motile sperm (D level) (Figure S2B), suggesting that a slightly alkaline environment optimally promoted motility.

### 3.2. Transcriptomics Analysis

#### 3.2.1. RNA-Seq Summary, Identification of DEGs, and PPI Network

**Figure 2:**
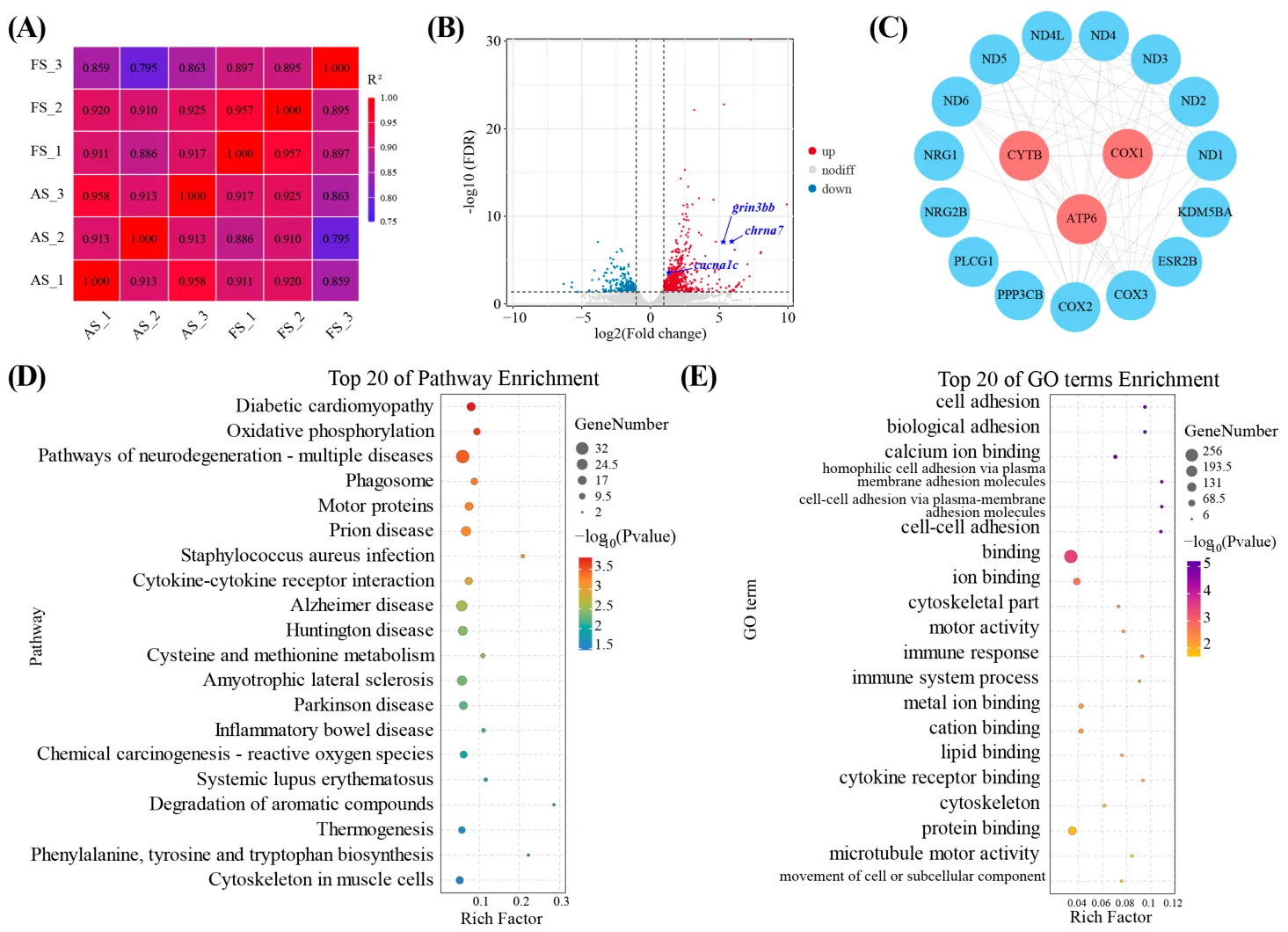
Transcriptomics analysis. (**A**) Pearson correlation coefficients. (**B**) Volcano plot of differentially expressed genes (DEGs) between activated sperm (AS) and fresh sperm (FS). (**C**) Protein–protein interaction (PPI) network of DEGs with a combined interaction score > 900, with red ellipses marking hub proteins and blue ellipses indicating interacting partners. (**D**) KEGG enrichment analysis. (**E**) GO enrichment analysis.

RNA-seq generated 264,432,676 raw reads from six sperm samples, yielding 245,324,144 clean reads (92.77%) after filtering (Table S1). Clean bases ranged from 6.02 to 6.32 Gbp, with GC contents of 50.23–51.41% and Q20/Q30 values above 97% and 92%, respectively. These results showed high sequencing quality. Reads mapped to the *S. chuatsi* reference genome at rates ranging from 66.97% to 78.92%, with 56.88–70.95% uniquely aligned. Transcriptome assembly identified 23,673 genes, including 22,630 known and 1043 novel genes. Biological replicates exhibited strong concordance within both FS and AS groups (Figure 2A). Differential expression analysis identified 855 DEGs between the AS and FS group, including 625 up-regulated and 230 down-regulated genes (Figure 2B; Supplementary Data S2). Several ion channel associated genes, including *cacna1c*, *grin3bb*, and *chrna7*, exhibited significant expression changes. PPI network analysis identified three hub mitochondrial genes (GYTB, COX1, and ATP6) (Figure 2C).

#### 3.2.2. KEGG and GO Enrichment Analyses of DEGs

KEGG enrichment analysis identified 25 significantly enriched pathways (*p* < 0.05) in the AS vs. FS comparison (Figure 2D; Supplementary Data S2). These pathways were mainly related to metabolism, cellular processes, organismal systems, and environmental information processing. Up-regulated DEGs were significantly enriched in oxidative phosphorylation (Figure S3A), whereas down-regulated DEGs were significantly enriched in PPAR signaling pathway (Figure S3B), suggesting that energy metabolism plays a critical role in sperm activation and movement.

GO enrichment analysis revealed 60 significantly enriched GO terms (*p* < 0.05), including 28 biological process (BP), 8 cellular component (CC), and 24 molecular function (MF) categories (Figure 2E; Supplementary Data S2). Up-regulated DEGs were significantly enriched in ion binding (GO:0043167), ATP binding (GO:0005524), and lipid binding (GO:0008289) (Figure S3C), whereas down-regulated DEGs were significantly enriched in regulation of metabolic process (GO:0019222) and signaling (GO:0023052) (Figure S3D). These findings indicate the importance of metabolic regulation and signal transduction after sperm activation.

### 3.3. Proteomics Analysis

#### 3.3.1. LC-MS/MS Summary, Identification of DEPs, and PPI Network

**Figure 3:**
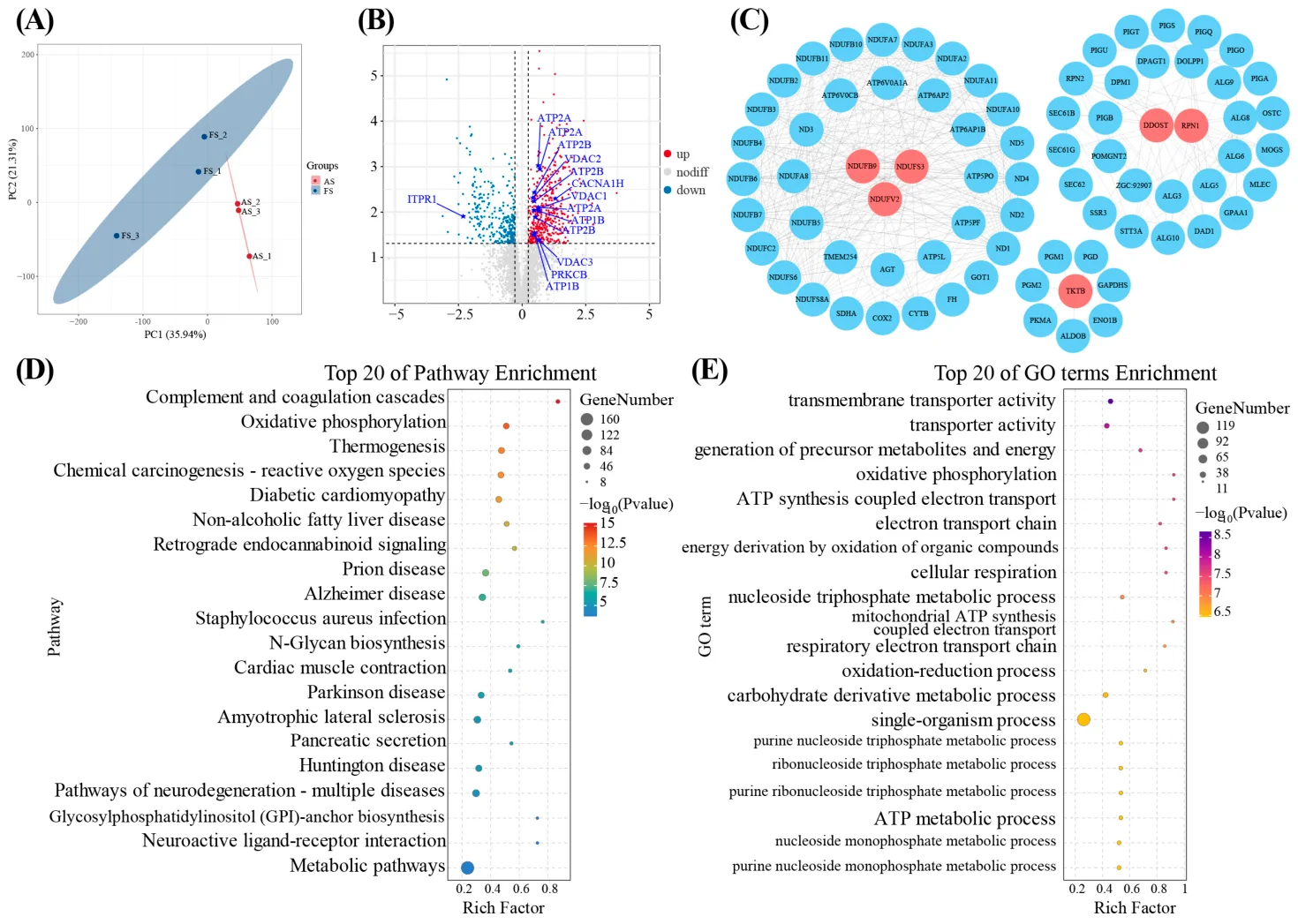
Proteomics analysis. (**A**) Principal component analysis (PCA). (**B**) Volcano plot showing differentially expressed proteins (DEPs) between AS and FS groups. (**C**) PPI network of DEPs with a combined interaction score > 900, with red ellipses marking hub proteins and blue ellipses indicating interacting partners. (**D**) KEGG enrichment analysis. (**E**) GO enrichment analysis.

Proteomic profiling identified 4037 proteins from 39,611 peptides. Functional annotation across Clusters of Orthologous Groups of proteins (KOG/COG), KEGG, GO, Trans_Factor, InterPro (IPR), and Subcellular databases showed 25 proteins consistently annotated in all datasets. PCA clearly distinguished AS from FS samples, suggesting marked proteomic differences between activated and fresh sperm (Figure 3A). A total of 752 DEPs were identified between two groups, including 297 up-regulated and 455 down-regulated proteins (Figure 3B; Supplementary Data S3). Ion channel and ion transport related proteins, including ITPR1, ATP2A, ATP2B, VDAC2, CACNA1H, VDAC1, ATP1B, VDAC3, and PRKCB, were differentially expressed. PPI network analysis identified six hub proteins, including NDUFB9, NDUFS3, NDUFV2, DDOST, RPN1, and TKTB (Figure 3C).

#### 3.3.2. KEGG and GO Enrichment Analyses of DEPs

KEGG pathway analysis revealed 62 significantly enriched pathways (*p* < 0.05) in the AS vs. FS comparison (Figure 3D; Supplementary Data S3). These pathways were primarily associated with cellular processes, environmental information processing, metabolism, and organismal systems. Up-regulated DEPs were significantly enriched in oxidative phosphorylation, metabolic pathways, cAMP signaling pathway, and calcium signaling pathway (Figure S4A), while down-regulated DEPs were significantly enriched in glycolysis/gluconeogenesis and biosynthesis of amino acids (Figure S4B). These results highlighted the critical roles of energy metabolism and ion signaling in regulating sperm activation and motility.

GO enrichment analysis identified 172 significantly enriched GO terms (*p* < 0.05), including 112 BP, 10 CC, and 50 MF (Figure 3E; Supplementary Data S3). Up-regulated DEPs were significantly enriched in oxidative phosphorylation (GO:0006119), ATP metabolic process (GO:0046034), ion transport (GO:0006811), cation transport (GO:0006812), and proton transport (GO:0015992) (Figure S4C), whereas down-regulated DEPs were significantly enriched in glycolytic process (GO:0006096) and carbohydrate metabolic process (GO:0005975) (Figure S4D), indicating that metabolic processes and ion transporters are pivotal for sperm activation and movement.

### 3.4. Integration of Transcriptomics and Proteomics Analyses

**Figure 4:**
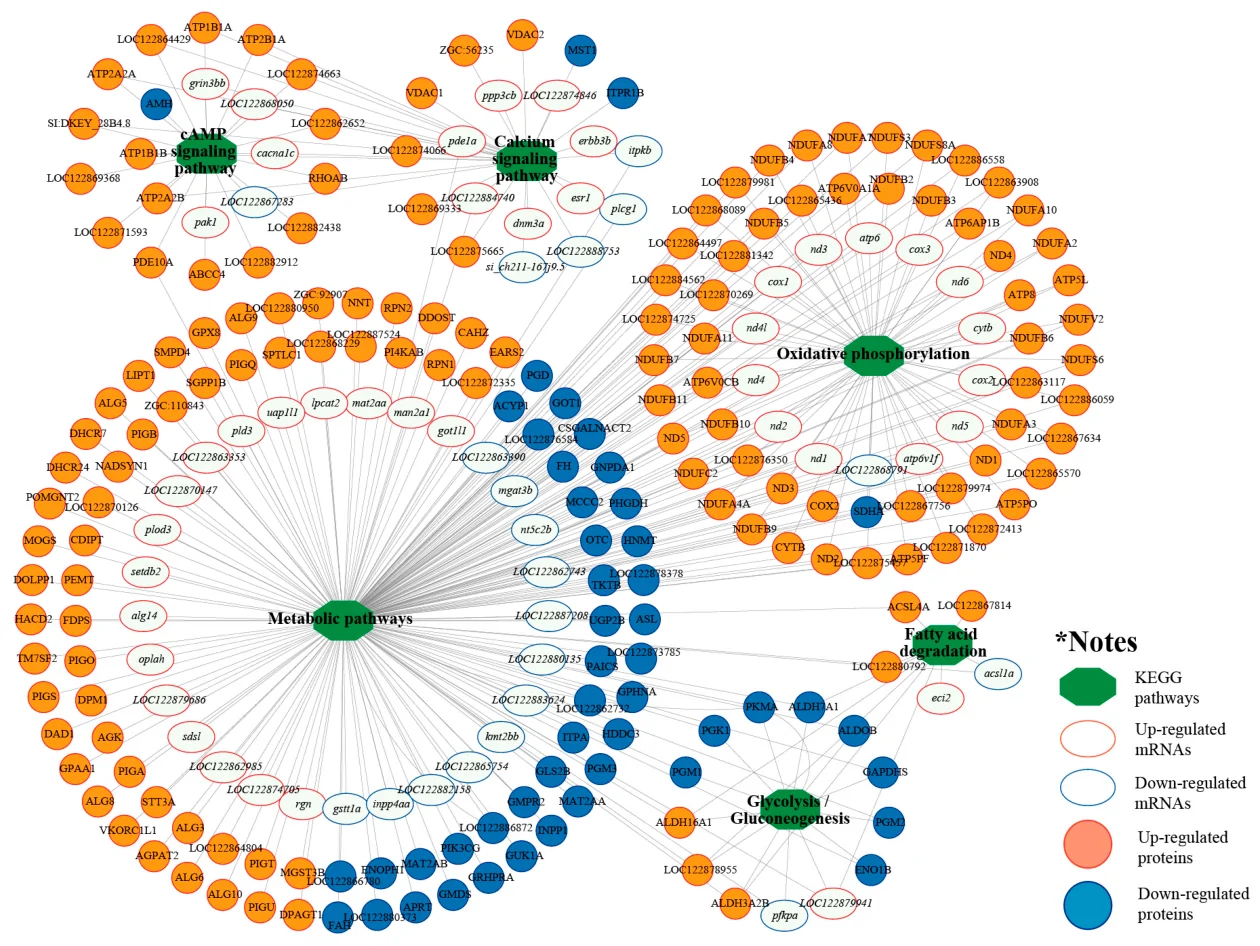
mRNA-pathway-protein network analysis. The pathway–DEGs–DEPs network included six key KEGG pathways, 64 DEGs, and 185 DEPs.

An mRNA–pathway–protein network was constructed using six key KEGG pathways, 64 DEGs, and 185 DEPs (Figure 4). Integration of transcriptomic and proteomic datasets identified coordinated changes across multiple biological pathways. Among these, proteins ATP2A, ATP1B, and ATP2B were associated with cAMP signaling pathways and calcium signaling pathways, while gene *pfkpa* and *LOC122879941* were together involved in glycolysis/gluconeogenesis and metabolic pathways.

**Figure 5:**
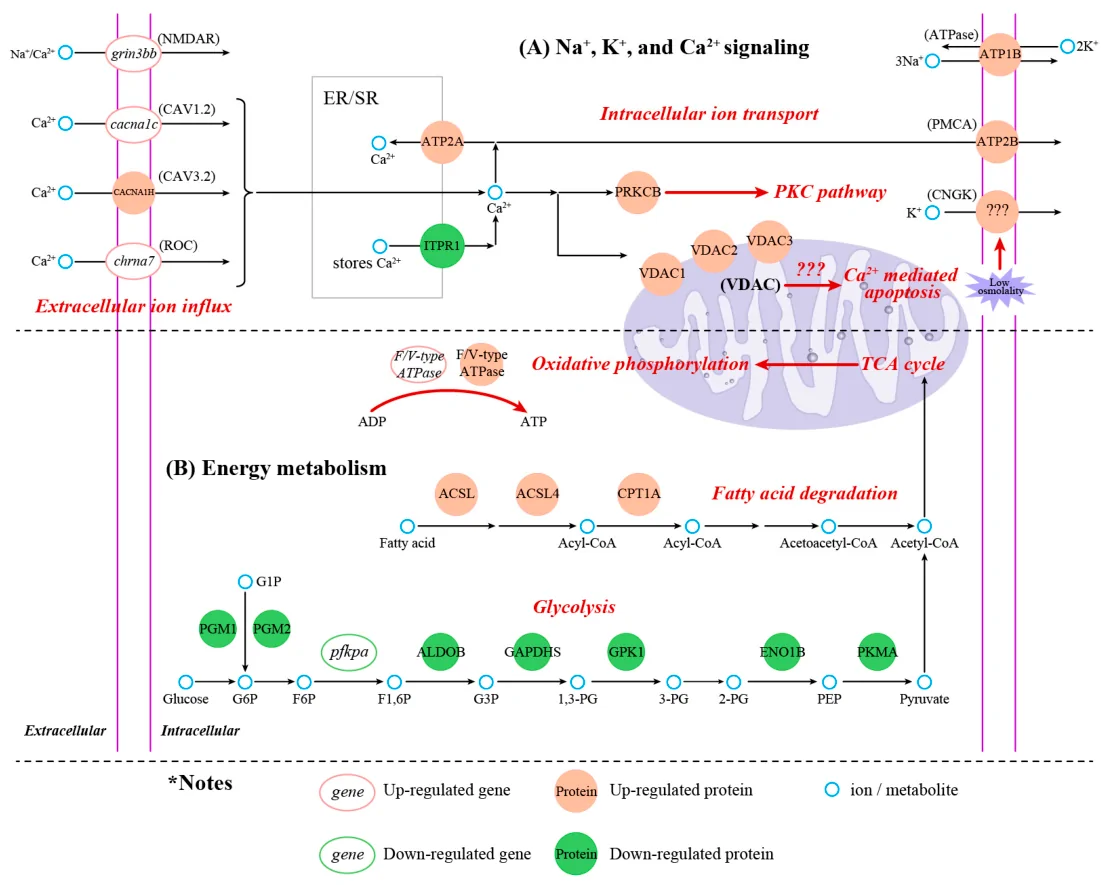
A putative transcriptomic and proteomic network revealing the molecular mechanisms underlying sperm activation and movement in mandarin fish. Transcriptomic and proteomic expression related to (**A**) Na^+^, K^+^, and Ca^2+^ signaling and (**B**) energy metabolism.

Integrative transcriptomics and proteomics identified six genes and 35 proteins associated with Na^+^, K^+^, and Ca^2+^ signaling and energy metabolism as key molecules of sperm activation and movement in mandarin fish. After sperm activation, genes related to extracellular Na^+^/Ca^2+^ influx (*grin3bb*), extracellular Ca^2+^ influx (*cacna1c*, *chrna7*), and oxidative phosphorylation (*atp6*, *atp6v1f*) were up-regulated, whereas glycolysis related gene (*pfkpa*) was down-regulated (Table S2). Protein expression patterns showed increased expression of factors involved in extracellular K^+^ influx (ATP1B), extracellular Ca^2+^ influx (CACNA1H), intracellular Ca^2+^ efflux (ATP2B), intracellular Ca^2+^ store (ATP2A), PKC pathway (PRKCB), Ca^2+^ mediated apoptosis (VDAC1, VDAC2, VDAC3), fatty acid degradation (ACSL, ACSL4, CPT1A), and oxidative phosphorylation (ATPeF0F6, ATPeF0D, ATPeF0O, ATPeFG, ATPeF08, ATPeV0D, ATPeVS1, ATPeV0A, ATPeV0C) (Figure S5), alongside decreased expression of glycolysis related proteins (PGM1, PGM2, ALDOB, GAPDHS, PGK1, ENO1B, PKMA) (Table S3). A putative transcriptomic and proteomic network was therefore established to reveal molecular mechanisms underlying sperm activation and movement in mandarin fish (Figure 5).

## 4. Discussion

### 4.1. Effects of Osmolality and Ions

Osmolality plays a critical role in teleost sperm activation, as sperm remain immotile in seminal plasma and are activated upon osmotic shifts [33,34]. In most freshwater fish, sperm activation and motility initiation rely on relatively low osmolality [2,35]. In the present study, mandarin fish sperm demonstrated strong activation at osmolalities of 0–150 mOsm/kg (motility > 70%), markedly lower than the seminal plasma osmolality (367–380 mOsm/kg). Similar observations have been reported in other freshwater fishes, where sperm activation is achieved at osmolalities below that of seminal plasma. In goldfish (*Carassius auratus*), sperm with a seminal plasma osmolality of about 317 mOsm/kg exhibit good activation across 100–200 mOsm/kg [36]. Sperm of Atlantic halibut (*Hippoglossus hippoglossus*, with seminal plasma osmolality of about 286 mOsm/kg) show high motility at 60–140 mOsm/kg [37]. Sperm of Java carp (*Puntius javanicus*) and catfish (*Clarias batrachus*) maintain strong motility at 10–100 mOsm/kg [6], while in bluegill (*Lepomis macrochirus*), with seminal plasma osmolality of approximately 290 mOsm/kg, high motility is observed at 50–180 mOsm/kg [11]. These results suggest that mandarin fish exhibit a characteristic freshwater pattern, similar to other freshwater fishes, with sperm activation initiated below seminal plasma osmolality and relatively high motility preserved across 0–150 mOsm/kg. After sperm activation, some marine fishes, such as sterlet (*Acipenser ruthenus*) [38] and Persian sturgeon (*Acipenser persicus*) [39], often sustain motility for more than 300 s. However, spermatozoa of most freshwater fish typically remain motile for less than 60 s [4]. Our results also showed that mandarin fish sperm motility decreased to below 30% after activation 30 s. Although relatively high osmolality (150–200 mOsm/kg) helped maintain motility, it reduced initial motility, suggesting limited energy availability in freshwater fish spermatozoa [2,4].

Freshwater fish spermatozoa are maintained in a quiescent or unactivated state within seminal plasma characterized by specific ionic compositions, including Na^+^ concentrations of approximately 75–131 mM, K^+^ concentrations of 10–82 mM, and Ca^2+^ concentrations of 0.7–2.4 mM [36,40,41]. Once released into hypoosmotic ambient freshwater, spermatozoa of most freshwater fish exhibit pronounced intracellular ion efflux, which lowers intracellular K^+^ concentration and further induces plasma membrane hyperpolarization, a key physiological stimulus responsible for freshwater fish sperm activation [2,7]. In this study, mandarin fish sperm were successfully activated in media with low concentrations of Na^+^ or K^+^ (≤25 mM) and exhibited high motility, suggesting that relatively low ionic strength facilitates sperm activation and movement. Similar findings, in which low concentrations of Na^+^ or K^+^ in activation solutions promote sperm motility have also been reported in several freshwater fish species, including common carp (*Cyprinus carpio* L.) [37], Java carp [6], catfish [6], and bluegill [11]. Na^+^ activation of the sperm-specific Na^+^/H^+^ exchanger (sNHE) promotes Na^+^ influx and H^+^ extrusion, leading to cytoplasmic alkalinization that enhances dynein-driven flagellar beating and sperm motility via increased dynein ATPase activity [42,43]. K^+^ influx via Na^+^/K^+^-ATPase and K^+^-selective channels promotes stable sperm membrane potential and maintains intracellular K^+^ homeostasis [44,45]. Previous studies have shown that high Na^+^ or K^+^ concentrations in activation media inhibit sperm activation and motility in some freshwater fishes [7,33]. Our results further showed that sperm motility in mandarin fish decreased progressively with increasing Na^+^ or K^+^ concentrations. Collectively, these findings suggest that low concentrations of Na^+^ or K^+^ promote sperm activation and movement in most freshwater fishes, whereas high ion concentrations inhibit these processes.

Ca^2+^ mediated signal transduction is critical for sperm activation and motility in most teleosts [1]. In most freshwater fishes, hypo-osmotic conditions induce intracellular K^+^ efflux and membrane hyperpolarization, leading to extracellular Ca^2+^ influx through membrane channels that activates flagellar Ca^2+^/calmodulin-dependent proteins required for sperm motility [2]. Notably, our physiological analysis suggested that Ca^2+^ inhibited mandarin fish sperm motility after activation. Similar observations have also been reported in sea urchin (*Tripneustes gratilla*) [46] and other freshwater fishes, such as Java carp [6] and bluegill [11], where Ca^2+^ supplementation in activation media suppresses sperm motility. Previous studies have shown that sperm quiescence results from Ca^2+^-induced inhibition of microtubule sliding, leading to suppression of asymmetric oscillatory beating [46]. Elevated intracellular Ca^2+^ may regulate sperm function via mitochondrial apoptotic pathways, such as mitochondrial voltage-dependent anion channel (VDAC)-mediated Ca^2+^-dependent apoptosis [47,48]. Additionally, Ca^2+^-activated calpain-1 cleaves cytoskeletal and regulatory proteins, triggering apoptotic signaling cascades [49,50], which may ultimately lead to sperm apoptosis and motility impairment. Thus, Ca^2+^ inhibits sperm motility in mandarin fish, and its molecular regulatory mechanisms are likely highly complex. Nevertheless, low cytoplasmic Ca^2+^ levels are inhibitory to sperm motility, whereas further reductions may permit or even promote motility [11].

### 4.2. Transcriptomic and Proteomic Regulatory Network

#### 4.2.1. Na^+^, K^+^, and Ca^2+^ Signaling

Under hypo-osmotic conditions, a reduction in extracellular K^+^ concentration promotes K^+^ efflux through pH-sensitive cyclic nucleotide-gated K^+^ channels (e.g., CNGK), resulting in sperm membrane hyperpolarization, with modulation by stretch-activated ion channels [2]. In zebrafish, sperm motility initiation is triggered by low external osmolality, which drives K^+^ efflux through K^+^-selective cyclic nucleotide-gated (CNGK) channels and results in membrane hyperpolarization [43,51]. The resulting hyperpolarization activates HCNL1-dependent proton influx, forming a pH-regulated negative feedback loop that regulates sperm activation and movement [51]. In our study, the *grin3bb* gene (Log2FC = 5.33) and ATP1B protein (FC = 1.59) were significantly up-regulated in mandarin fish. The *grin3bb* gene, encoding a subunit of N-methyl-D-aspartate receptors (NMDARs), functions as ligand-gated ion channels that permit Na^+^ and Ca^2+^ influx upon agonist binding [52]. ATP1B mediates extracellular K^+^ influx through Na^+^/K^+^-ATPase and K^+^-selective channels [53]. Therefore, in mandarin fish, hypo-osmotic conditions together with a Na^+^/K^+^ activation medium may enhance sperm motility by maintaining ionic homeostasis through Na^+^ and K^+^ influx via sperm ion channels.

The hypo-osmotic signal is accompanied by elevations in intracellular Ca^2+^ and pH, and Ca^2+^ influx may further regulate PKC signaling and the activity of Ca^2+^/calmodulin-dependent proteins associated with the axoneme [2]. In this study, our transcriptomic and proteomic profiling identified increased expression of *cacna1c* (Log2FC = 1.37) and *chrna7* (Log2FC = 5.95), together with elevated abundance of CACNA1H (FC = 2.50) and PRKCB (FC = 1.60). Voltage-dependent calcium channels (VDCCs) contribute to cytosolic Ca^2+^ increases by mediating extracellular Ca^2+^ entry, triggering Ca^2+^-dependent signaling cascades [54,55]. The α7 nicotinic acetylcholine receptor (CHRNA7) promotes Ca^2+^ entry into spermatozoa via its high Ca^2+^ permeability, membrane depolarization, and mobilization of intracellular Ca^2+^ stores, thereby enhancing flagellar motility and energy metabolism [56,57]. Elevated intracellular Ca^2+^ promotes PKCβ (PRKCB) recruitment and activation at the membrane, which phosphorylates axonemal proteins and metabolic enzymes, orchestrating the initiation and maintenance of sperm motility [58]. Thus, the coordinated up-regulation of NMDAR, VDCC, and CHRNA7 associated genes and proteins may facilitate extracellular Ca^2+^ influx and activate the PKC signaling pathway, thereby promoting sperm activation and movement in mandarin fish. In addition, we detected significant down-regulation of the itpr1 gene and up-regulation of the ATP2A (FC = 1.57) and ATP2B (FC = 1.41) proteins. The *itpr1* gene, which mediates Ca^2+^ release from ER/SR-like intracellular stores [59], was significantly down-regulated, indicating a reduction in Ca^2+^ mobilization from internal stores. In contrast, the up-regulation of ATP2A, encoding ER/SR Ca^2+^-ATPase (SERCA), promotes ATP-dependent Ca^2+^ pumping into the ER/SR [60], maintaining cytosolic Ca^2+^ homeostasis and intracellular Ca^2+^ signaling cycles. Concurrently, up-regulated ATP2B (plasma membrane Ca^2+^-ATPase, PMCA) mediates active extrusion of Ca^2+^ across the plasma membrane [61,62], thereby preserving cellular Ca^2+^ homeostasis. Notably, proteins (VDAC1, VDAC1, and VDAC3) associated with VDACs were significantly up-regulated. Given the role of VDAC in Ca^2+^ mediated apoptosis [47,48,63], its increased expression may be linked to the inhibitory effects of Ca^2+^ activation media on sperm motility in mandarin fish. Nevertheless, the mechanisms by which Ca^2+^ suppresses fish sperm activation and movement remain largely unresolved. Further investigation is required to identify the molecular mechanisms underlying this inhibitory process.

#### 4.2.2. Energy Metabolism

Energy metabolism is essential for fish sperm activation and movement, providing the ATP required to initiate and sustain flagellar beating, thus ensuring successful fertilization [64,65]. Glucose serves as a key energy substrate during sperm storage, but upon activation, glycolytic activity gradually declines, accompanied by a metabolic shift toward alternative energy production pathways [65,66]. In the present study, transcriptomic and proteomic data showed that the gene (*pfkpa*) and several proteins (PGM1, PGM2, ALDOB, GAPDHS, PGK1, ENO1B, PKMA) involved in glycolysis were significantly down-regulated after sperm activation, indicating the suppression of glycolytic metabolism. A similar suppression of glycolysis during sperm movement has also been observed in Nile tilapia [67]. Energy production shifts from reliance on glycogenolysis and glycolysis before activation to dependence on pyruvate metabolism and fatty acid β-oxidation after activation, supporting the energetic demands of sustained sperm motility [67]. KEGG enrichment analysis identified oxidative phosphorylation as one of the most significantly enriched pathways in both the transcriptomic and proteomic datasets (Figure S5), highlighting the important regulation of mitochondrial energy metabolism. Our transcriptomic and proteomic analyses revealed significant up-regulation of two genes and ten proteins involved in F/V-type ATPase-dependent oxidative phosphorylation, suggesting that enhanced ATP production occurs after sperm activation to meet the energetic demands of mandarin fish sperm movement. In addition, proteins involved in fatty acid metabolism, including ACSL (FC = 1.74), ACSL4 (FC = 1.53), and CPT1A (FC = 1.63), were significantly up-regulated after sperm activation, suggesting increased utilization of fatty acids as an energy source through fatty acid degradation. Several teleost species, including Kara whitefish (*Chalcalburnus chalcoides*) [68], rainbow trout (*Oncorhynchus mykiss*) [64,68], and common carp [69,70], have also been reported to rely on fatty acid metabolism and oxidative phosphorylation to maintain sperm movement. Collectively, these findings suggest that sperm activation and movement in mandarin fish are accompanied by a metabolic shift from glycolysis toward fatty acid degradation and mitochondrial oxidative phosphorylation, which ensures efficient ATP production to sustain sperm motility.

### 4.3. Implications for Aquaculture, Limitations, and Prospects

Based on these findings, our physiological analysis suggests that in the artificial fertilization practices of mandarin fish, 1‰–1.5‰ NaCl, KCl, or their mixtures (total concentration < 25 mM) can serve as effective activation media for sperm activation and fertilization in mandarin fish. Meanwhile, high concentrations of Ca^2+^ in activation media should be avoided, as only trace levels (Ca^2+^ < 1:500 molar ratio, <0.06 mM) appear to have negligible effects on sperm activation and motility. In addition, other divalent cations, such as Mg^2+^, Mn^2+^, Sr^2+^, Cd^2+^, and Ba^2+^, may also affect sperm activation and motility in freshwater fishes [11]. Our transcriptomic and proteomic analyses identified six DEGs and 35 DEPs as key molecules associated with Na^+^, K^+^, and Ca^2+^ signaling and energy metabolism. However, these omics results are preliminary and provide a putative model for sperm activation and movement mechanisms. These key molecules, ion channels, and biological processes require further validation using electrophysiology, immunocytochemistry, in situ hybridization, and Western blot analyses [43,51]. In addition, because the omics analyses were performed at a single post-activation time point (60 s), future studies should incorporate multiple sampling time points and higher-resolution approaches to characterize the dynamic transcriptomic and proteomic changes throughout sperm activation.

## 5. Conclusions

This study integrates physiological and multi-omic approaches to delineate the mechanisms underlying sperm activation and movement in mandarin fish. Low osmolality (≤50 mOsm/kg) promotes sperm activation in an osmolality-dependent manner, with motility decreasing as osmolality increases. Na^+^ and K^+^ activation media enhance motility, whereas Ca^2+^ supplementation suppresses activation. Consequently, we advise restricting Ca^2+^ concentrations during artificial fertilization to below a 1:500 molar ratio (<0.06 mM) to avoid significant effects on motility. Transcriptomic and proteomic analyses identify regulatory networks associated with Na^+^, K^+^, and Ca^2+^ signaling and energy metabolism. Up-regulation of ion channel-related genes (*grin3bb*, *cacna1c*, and *chrna7*) and proteins (ATP1B, ATP2B, ATP2A, CACNA1H, and PRKCB) contributes to the maintenance of intracellular ionic homeostasis. Increased VDAC expression may promote Ca^2+^-mediated mitochondrial apoptosis, potentially leading to sperm apoptosis and reduced motility. A metabolic shift from glycolysis toward fatty acid degradation and oxidative phosphorylation occurs after activation. Our study identifies key physiological determinants and provides insights into the putative molecular mechanisms underlying sperm activation and movement in mandarin fish, providing a basis for optimizing artificial fertilization.

## Data Availability

All transcriptomic and proteomic raw data are deposited in the NCBI Sequence Read Archive (SRA) database (accession number PRJNA1472532) and National Genomics Data Center (NGDC, China) OMIX database (https://ngdc.cncb.ac.cn/, accession number OMIX017197), respectively.

## Supplementary Materials

The following supporting information can be downloaded at https://www.mdpi.com/article/10.3390/ani16172762/s1: Figure S1: Effect of NaCl and KCl on sperm motility; Figure S2: Effect of pH on sperm motility; Figure S3: Enrichment analysis of up-regulated and down-regulated DEGs; Figure S4: Enrichment analysis of up-regulated and down-regulated DEPs; Figure S5: KEGG pathway in oxidative phosphorylation for DEGs and DEPs; Table S1: Summary of RNA-seq; Table S2: Key genes associated with Na^+^ and Ca^2+^ signaling and energy metabolism; Table S3: Key proteins associated with K^+^ and Ca^2+^ signaling and energy metabolism; Supplementary Data S1: Sperm motility of physiological analysis; Supplementary Data S2: Transcriptomics; Supplementary Data S3: Proteomics.

## Abbreviations

The following abbreviations are used in this manuscript:

*atp6*	F-type H^+^-transporting ATPase subunit a (ATP6)
*atp6v1f*	V-type H^+^-transporting ATPase subunit F
*cacna1c*	voltage-dependent calcium channel L type alpha-1C
*chrna7*	nicotinic acetylcholine receptor alpha-7
*grin3bb*	glutamate receptor ionotropic, NMDA 3B
*pfkpa*	6-phosphofructokinase 1
ACSL	long-chain-fatty-acid---CoA ligase
ACSL4A	long-chain-fatty-acid---CoA ligase 4
ALDOB	fructose bisphosphate aldolase B
ATP1B	sodium/potassium-transporting ATPase subunit beta
ATP2A	P-type Ca^2+^ transporter type 2A
ATP2B	P-type Ca^2+^ transporter type 2B
ATPeF08	F-type H^+^-transporting ATPase subunit 8
ATPeF0D	F-type H^+^-transporting ATPase subunit d
ATPeF0F6	F-type H^+^-transporting ATPase subunit 6
ATPeF0O	F-type H^+^-transporting ATPase subunit O
ATPeFG	F-type H^+^-transporting ATPase subunit g
ATPeV0A	V-type H^+^-transporting ATPase subunit a
ATPeV0C	V-type H^+^-transporting ATPase subunit cb
ATPeV0D	V-type H^+^-transporting ATPase subunit d
ATPeVS1	V-type H^+^-transporting ATPase S1 subunit
CACNA1H	voltage-dependent calcium channel T type alpha-1H (CAV3.2)
CPT1A	carnitine O-palmitoyltransferase 1, liver isoform
ENO1B	enolase 1b, (alpha) isoform X1
GAPDHS	glyceraldehyde-3-phosphate dehydrogenase, spermatogenic
ITPR1	inositol 1,4,5-triphosphate receptor type 1
PGK1	phosphoglycerate kinase 1
PGM1	phosphoglucomutase 1
PGM2	phosphoglucomutase 2
PKMA	pyruvate kinase PKM isoform X2
PKMA	pyruvate kinase PKM isoform X1
PRKCB	protein kinase C beta type isoform X1
VDAC1	voltage-dependent anion channel protein 1
VDAC2	voltage-dependent anion channel protein 2
VDAC3	voltage-dependent anion channel protein 3
