# Supplementary material for: Integrating Physiology and Multi-Omics Reveals Mechanisms Underlying Sperm Activation and Movement in Mandarin Fish (Siniperca chuatsi)
Source: Animals (Basel). 2026 Sep 2;16(17):2762. doi: 10.3390/ani16172762 (PMC13565002; doi:10.3390/ani16172762)
Supplement: Supplementary file 1 [file animals-16-02762-s001.zip › Supplementary_Figure.pdf]

Supplementary Figures

Integrating physiology and multi-omics reveals mechanisms underlying sperm activation and movement in mandarin fish (*Siniperca chuatsi*)

Qinghua Wang [1], Yuxin Zhang [1], Zhong Huang [3], Weiwei Zhang [1], Yingxin Wu [1], Jiajie Li [1], Yizheng Zhang [1], Lu Li [1], Zhiming Zhu [1,2,*] and Zining Meng [1,2,*]

[1] School of Life Sciences, State Key Laboratory of Biocontrol / Guangdong Core Germplasm Bank for Marine Economic Animals, Southern Marine Science and Engineering Guangdong Laboratory (Zhuhai) / Guangdong Provincial Key Laboratory of Aquatic Economic Animals, Sun Yat-sen University, Guangzhou 510275, China; wangqh55@mail2.sysu.edu.cn (Q.H.W.); zhangyx558@mail2.sysu.edu.cn (Y.X.Z.); zhangww79@mail2.sysu.edu.cn (W.W.Z.); wuyx328@mail2.sysu.edu.cn (Y.X.W.); lijj379@mail2.sysu.edu.cn (J.J.L.); zhangyzh253@mail2.sysu.edu.cn (Y.Z.Z.); lilu95@mail2.sysu.edu.cn (L.L.)

[2] China-ASEAN Belt and Road Joint Laboratory on Mariculture Technology, Guangzhou 510275, China

[3] Shenzhen Base of South China Sea Fisheries Research Institute, Chinese Academy of Fishery Sciences, Shenzhen, China; huangzhongnhs@163.com (Z.H.)

* Correspondence: zhuzhiming@sml-zhuhai.cn (Z.M.Z.); mengzn@mail.sysu.edu.cn (Z.N.M.)

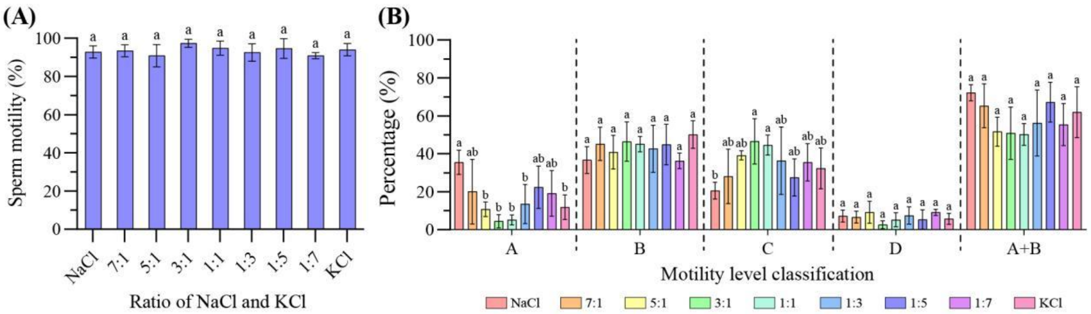

Figure S1. Effect of NaCl and KCl on sperm motility. (A) Sperm motility at 50 mOsm/kg and 10 s post-activation under varying NaCl and KCl combinations. Different lowercase letters represent significant differences among treatments (P < 0.05). (B) Motility level classification (A, B, C, and D) under the same conditions. Different lowercase letters indicate significant differences within each motility category (P < 0.05).

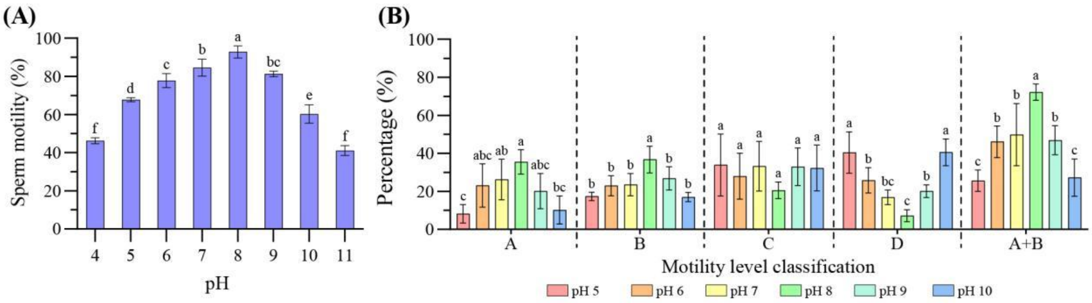

Figure S2. Effect of pH on sperm motility. (A) Sperm motility at 50 mOsm/kg and 10 s post-activation using NaCl-based activation medium under varying pH. Different lowercase letters represent significant differences among treatments (P < 0.05). (B) Motility level classification (A, B, C, and D) under the same conditions. Different lowercase letters indicate significant differences within each motility category (P < 0.05).

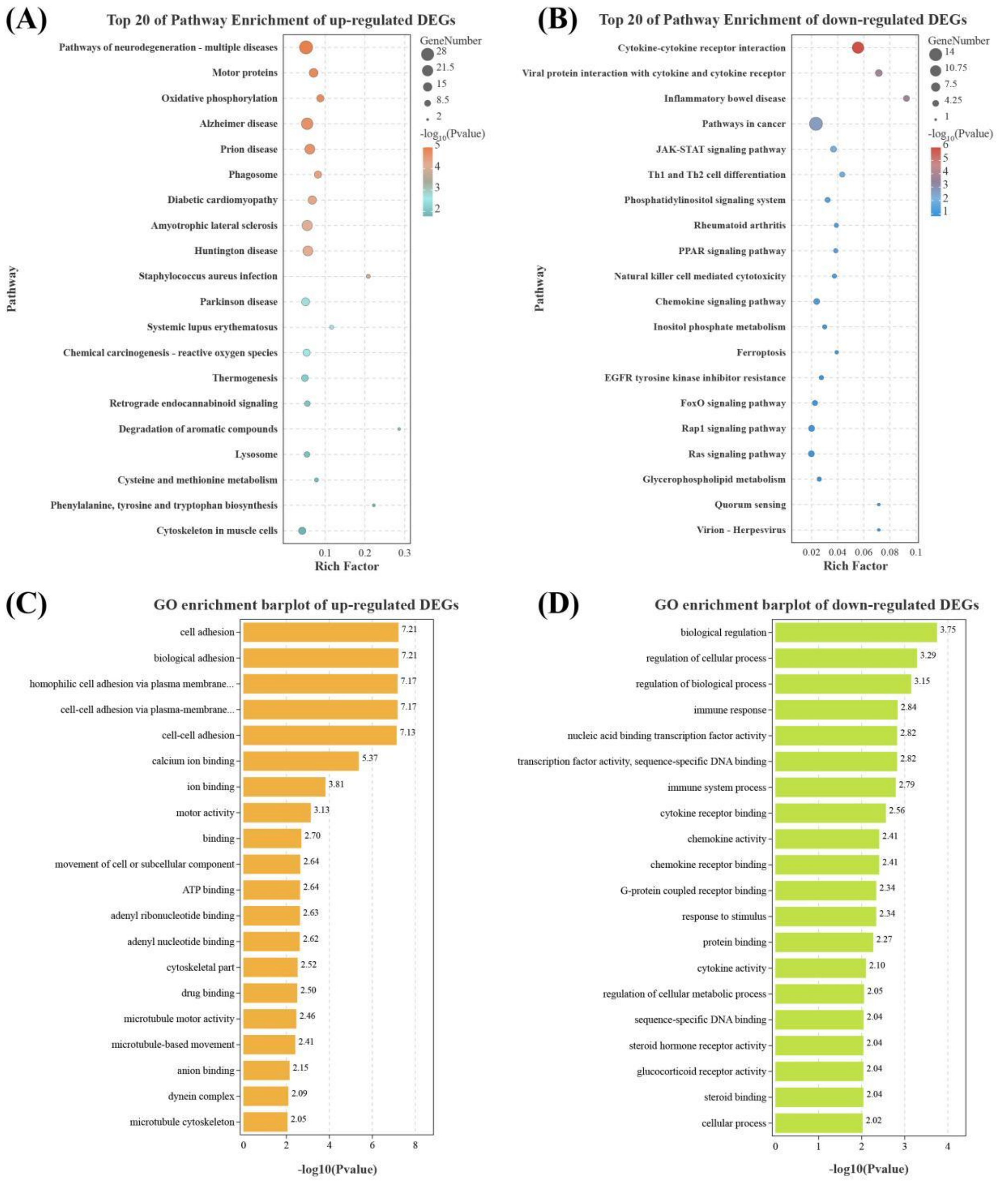

Figure S3. Enrichment analysis of up-regulated and down-regulated DEGs. KEGG enrichment analysis in AS vs. FS comparison of up-regulated (A) and down-regulated (B) DEGs. GO enrichment analysis in AS vs. FS comparison of up-regulated (C) and down-regulated (D) DEGs.

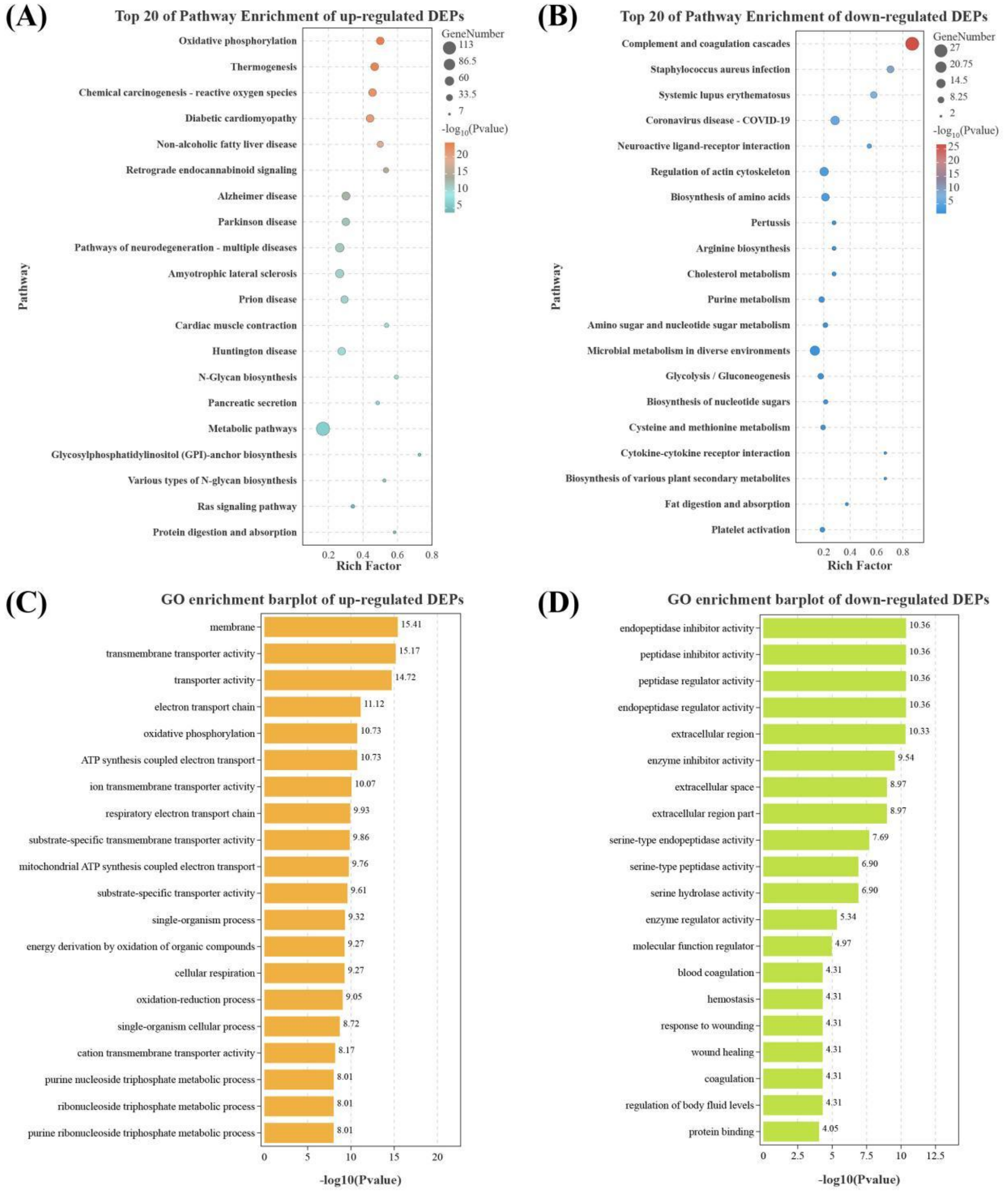

Figure S4. Enrichment analysis of up-regulated and down-regulated DEPs. KEGG enrichment analysis in AS vs. FS comparison of up-regulated (A) and down-regulated (B) DEPs. GO enrichment analysis in AS vs. FS comparison of up-regulated (C) and down-regulated (D) DEPs.

(A)

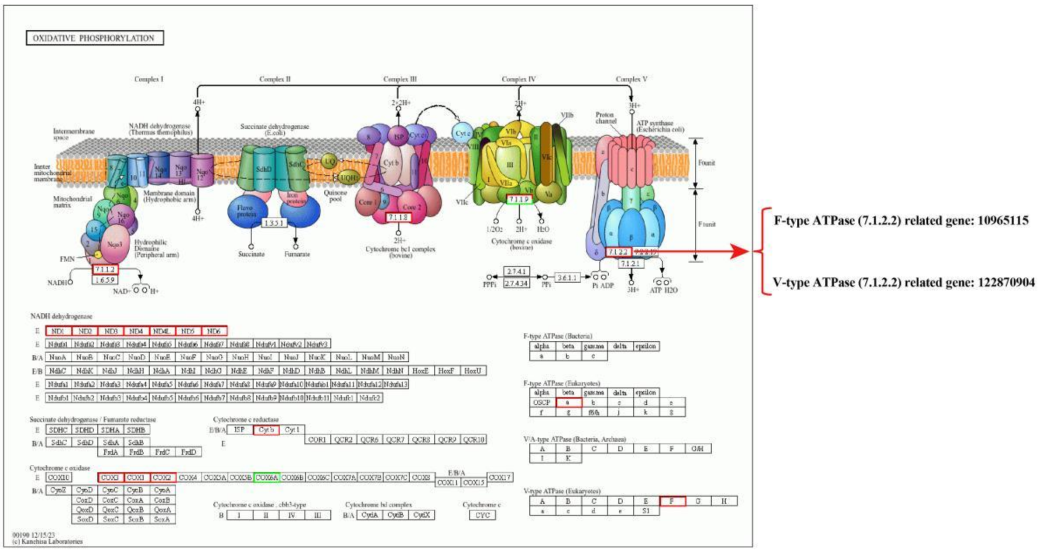

(B)

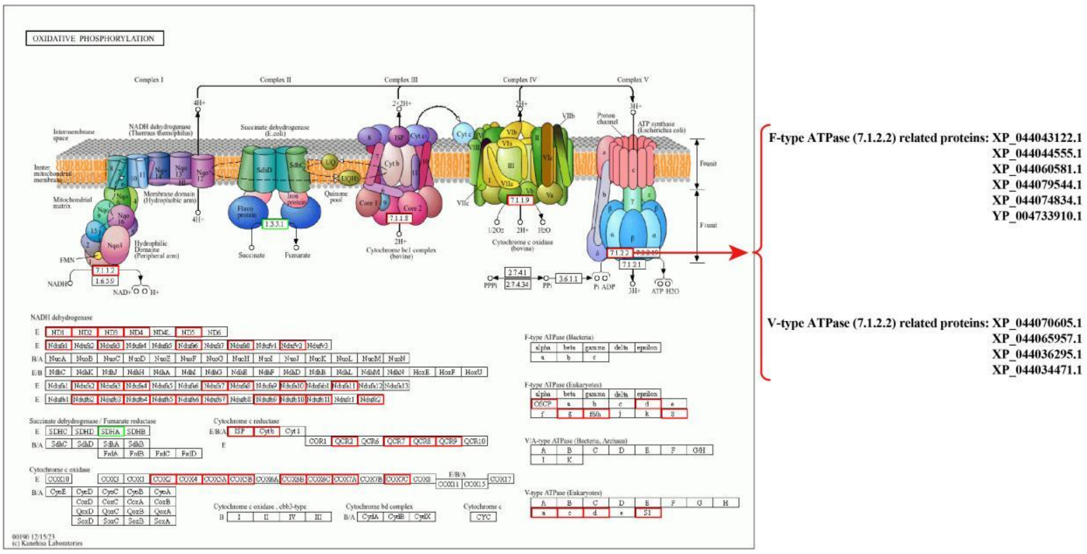

Figure S5. KEGG pathway in oxidative phosphorylation for DEGs (A) and DEPs (B).
