# Supplementary material for: Integrating Physiology and Multi-Omics Reveals Mechanisms Underlying Sperm Activation and Movement in Mandarin Fish (Siniperca chuatsi)
Source: Animals (Basel). 2026 Sep 2;16(17):2762. doi: 10.3390/ani16172762 (PMC13565002; doi:10.3390/ani16172762)
Supplement: Supplementary file 1 [file animals-16-02762-s001.zip › Supplementary_Table.pdf]

Supplementary Tables

Integrating physiology and multi-omics reveals mechanisms underlying sperm activation and movement in mandarin fish (*Siniperca chuatsi)*

Qinghua Wang [1], Yuxin Zhang [1], Zhong Huang [3], Weiwei Zhang [1], Yingxin Wu [1], Jiajie Li [1], Yizheng Zhang [1], Lu Li [1], Zhiming Zhu [1,2,*] and Zining Meng [1,2,*]

[1] School of Life Sciences, State Key Laboratory of Biocontrol / Guangdong Core Germplasm Bank for Marine Economic Animals, Southern Marine Science and Engineering Guangdong Laboratory (Zhuhai) / Guangdong Provincial Key Laboratory of Aquatic Economic Animals, Sun Yat-sen University, Guangzhou 510275, China; wangqh55@mail2.sysu.edu.cn (Q.H.W.); zhangyx558@mail2.sysu.edu.cn (Y.X.Z.); zhangww79@mail2.sysu.edu.cn (W.W.Z.); wuyx328@mail2.sysu.edu.cn (Y.X.W.); lijj379@mail2.sysu.edu.cn (J.J.L.); zhangyzh253@mail2.sysu.edu.cn (Y.Z.Z.); lilu95@mail2.sysu.edu.cn (L.L.)

[2] China-ASEAN Belt and Road Joint Laboratory on Mariculture Technology, Guangzhou 510275, China

[3] Shenzhen Base of South China Sea Fisheries Research Institute, Chinese Academy of Fishery Sciences, Shenzhen, China; huangzhongnhs@163.com (Z.H.)

* Correspondence: zhuzhiming@sml-zhuhai.cn (Z.M.Z.); mengzn@mail.sysu.edu.cn (Z.N.M.)

Table S1. Summary of RNA-seq.

Sample	Raw reads	Clean reads	Raw bases (Gbp)	Clean bases (Gbp)	GC (%)	Q20 (%)	Q30 (%)	Total mapped (%)	Unique mapped (%)
FS_1	46,183,628	42,163,580	6.93	6.32	50.51	96.97	92.32	72.59	65.99
FS_2	43,751,094	40,327,132	6.56	6.05	51.41	97.85	93.91	78.92	70.95
FS_3	44,692,706	41,150,690	6.70	6.17	50.67	97.32	93.2	73.71	66.45
AS_1	44,262,754	40,338,124	6.64	6.05	50.75	97.71	93.77	71.92	62.96
AS_2	43,685,884	40,138,028	6.55	6.02	50.61	97.59	93.58	68.75	60.16
AS_3	41,856,610	41,206,590	6.28	6.18	50.23	97.07	92.57	66.97	56.88

Table S2. Key genes associated with Na^+ and Ca^{2+} signaling and energy metabolism.

Gene ID	Name	Description	Log2FC	Regulation	Biological process/pathway	FS_FPKM	AS_FPKM
**Na^+ and Ca^{2+} signaling**							
122878298	*grin3bb*	glutamate receptor ionotropic, NMDA 3B	5.33	Up	Extracellular Na^+/Ca^{2+} influx	1.59	59.07
122871614	*cacna1c*	voltage-dependent calcium channel L type alpha-1C	1.37	Up	Extracellular Ca^{2+} influx	2,017.51	5,207.87
122874846	*chrna7*	nicotinic acetylcholine receptor alpha-7	5.95	Up	Extracellular Ca^{2+} influx	1.06	59.77
**Energy metabolism**							
122866679	*pfkpa*	6-phosphofructokinase 1	-1.04	Down	Glycolysis	347.42	170.39
10965115	*atp6*	F-type H+-transporting ATPase subunit a (ATP6)	1.86	Up	Oxidative phosphorylation	2,958.35	10,749.00
122870904	*atp6v1f*	V-type H+-transporting ATPase subunit F	1.04	Up	Oxidative phosphorylation	226.94	467.30

Table S3. Key proteins associated with K^+ and Ca^{2+} signaling and energy metabolism.

Protein ID	Name	Description	FC	Regulation	Biological process/pathway	FS_abundance	AS_abundance
**Na^+ and K^+ signaling**							
XP_044055315.1	ATP1B	sodium/potassium-transporting ATPase subunit beta	1.42	Up	Extracellular K^+ influx	521062	743854
XP_044043840.1	ATP1B	sodium/potassium-transporting ATPase subunit beta	1.59	Up	Extracellular K^+ influx	55894	89112
XP_044038174.1	CACNA1H	voltage-dependent calcium channel T type alpha-1H (CAV3.2)	2.50	Up	Extracellular Ca^{2+} influx	4897825	6879994
XP_044048776.1	ATP2B	P-type Ca^{2+} transporter type 2B	1.40	Up	Intracellular Ca^{2+} efflux	84823	119888
XP_044041733.1	ATP2B	P-type Ca^{2+} transporter type 2B	1.41	Up	Intracellular Ca^{2+} efflux	223541	323774
XP_044027775.1	ATP2B	P-type Ca^{2+} transporter type 2B	1.45	Up	Intracellular Ca^{2+} efflux	47398	74393
XP_044054090.1	ATP2A	P-type Ca^{2+} transporter type 2A	1.57	Up	Intracellular Ca^{2+} store	1434994	2253476
XP_044045569.1	ATP2A	P-type Ca^{2+} transporter type 2A	1.57	Up	Intracellular Ca^{2+} store	875617	1437623
XP_044029755.1	ATP2A	P-type Ca^{2+} transporter type 2A	1.64	Up	Intracellular Ca^{2+} store	129583	324435
XP_044022665.1	ITPR1	inositol 1,4,5-triphosphate receptor type 1	0.20	Down	ER/SR Ca2+ release	8370713	1705046
XP_044047528.1	PRKCB	protein kinase C beta type isoform X1	1.60	Up	PKC pathway	13471	21585
XP_044078981.1	VDAC1	voltage-dependent anion channel protein 1	1.40	Up	Ca^{2+} mediated apoptosis	175469	245885
XP_044069947.1	VDAC2	voltage-dependent anion channel protein 2	1.36	Up	Ca^{2+} mediated apoptosis	1062567	1445749
XP_044031258.1	VDAC3	voltage-dependent anion channel protein 3	1.38	Up	Ca^{2+} mediated apoptosis	58235	80264
**Energy metabolism**							
XP_044055997.1	PGM1	phosphoglucomutase 1	0.83	Down	Glycolysis	566384	470566
XP_044047110.1	PGM2	phosphoglucomutase 2	0.70	Down	Glycolysis	587389	408547
XP_044061318.1	ALDOB	fructose bisphosphate aldolase B	0.73	Down	Glycolysis	187785	136196
XP_044062571.1	GAPDHS	glyceraldehyde-3-phosphate dehydrogenase, spermatogenic	0.72	Down	Glycolysis	5140799	3691788
XP_044077607.1	PGK1	phosphoglycerate kinase 1	0.81	Down	Glycolysis	1864069	1505355
XP_044022029.1	ENO1B	enolase 1b	0.67	Down	Glycolysis	1246082	837379
XP_044060383.1	PKMA	pyruvate kinase PKM isoform X2	0.51	Down	Glycolysis	234882	119579
XP_044060372.1	PKMA	pyruvate kinase PKM isoform X1	0.81	Down	Glycolysis	1721949	1388534

XP_044062190.1	ACSL	long-chain-fatty-acid---CoA ligase	1.74	Up	Fatty acid degradation	93254	142864
XP_044062008.1	ACSL4	long-chain-fatty-acid---CoA ligase 4	1.53	Up	Fatty acid degradation	41276	71955
XP_044035017.1	CPT1A	carnitine O-palmitoyltransferase 1	1.63	Up	Fatty acid degradation	58916	96104
XP_044043122.1	ATPeF0F6	F-type H+-transporting ATPase subunit 6	1.34	Up	Oxidative phosphorylation	601958	805210
XP_044044555.1	ATPeF0D	F-type H+-transporting ATPase subunit d	1.44	Up	Oxidative phosphorylation	249358	358734
XP_044060581.1	ATPeF0O	F-type H+-transporting ATPase subunit O	1.46	Up	Oxidative phosphorylation	553341	806105
XP_044079544.1	ATPeFG	F-type H+-transporting ATPase subunit g	1.64	Up	Oxidative phosphorylation	1133303	1862643
XP_044074834.1	ATPeFG	F-type H+-transporting ATPase subunit g	1.76	Up	Oxidative phosphorylation	207178	363605
YP_004733910.1	ATPeF08	F-type H+-transporting ATPase subunit 8	3.23	Up	Oxidative phosphorylation	184345	594986
XP_044070605.1	ATPeV0D	V-type H+-transporting ATPase subunit d	2.12	Up	Oxidative phosphorylation	31947	67732
XP_044065957.1	ATPeVS1	V-type H+-transporting ATPase S1 subunit	3.30	Up	Oxidative phosphorylation	23890	78921
XP_044036295.1	ATPeV0A	V-type H+-transporting ATPase subunit a	3.48	Up	Oxidative phosphorylation	44566	155262
XP_044034471.1	ATPeV0C	V-type H+-transporting ATPase subunit cb	4.98	Up	Oxidative phosphorylation	4139	30889
